# Human Respiratory Coronaviruses Detected In Patients with Influenza-Like Illness in Arkansas, USA

**DOI:** 10.4172/2161-0517.S2-004

**Published:** 2014-03-26

**Authors:** Camila S Silva, Lisa B Mullis, Olavo Pereira, Linda J Saif, Anastasia Vlasova, Xuming Zhang, Randall J Owens, Dale Paulson, Deborah Taylor, Lia M Haynes, Marli P Azevedo

**Affiliations:** 1 Division of Microbiology, National Center for Toxicological Research, US Food and Drug Administration, USA; 2 Food Animal Health Research Program, Ohio Agricultural Research and Development Center, The Ohio State University, USA; 3 Department of Microbiology and Immunology, University of Arkansas for Medical Sciences, Little Rock, USA; 4 Public Health Laboratory, Arkansas Department of Health, Little Rock, USA; 5 Center for Biologics Evaluation and Research, US FDA, Rockville, USA; 6 National Center for Immunization and Respiratory Diseases, Division of Viral Diseases, Gastroenteritis and Respiratory Viruses Laboratory Branch, Centers for Disease Control and Prevention (CDC), USA

**Keywords:** Human respiratory coronaviruses, Molecular epidemiology, Influenza

## Abstract

Acute respiratory viruses often result in significant morbidity and mortality. The potential impact of human respiratory coronavirus (CoV) infections was underestimated until the severe acute respiratory syndrome (SARS-CoV) outbreak in 2003, which showed that new, highly pathogenic coronaviruses could be introduced to humans, highlighting the importance of monitoring the circulating coronaviruses. The use of sensitive molecular methods has contributed to the differential diagnosis of viruses circulating in humans. Our study aim was to investigate the molecular epidemiology of human CoV strains circulating in Arkansas, their genetic variability and their association with reported influenza-like symptoms. We analyzed 200 nasal swab samples, collected by the Arkansas Department of Health in 2010, for influenza diagnosis. All samples were from patients showing acute respiratory symptoms while testing negative for influenza. Samples were pre-screened, using a quantitative reverse transcriptase polymerase chain reaction (qRT-PCR) multiprobe for coronavirus, and subjected to confirmatory pancoronavirus and/or strain-specific reverse transcriptase (RT)-PCR followed by sequence analysis. Seventy-nine samples (39.5%) were positive by qRT-PCR and 35 samples (17.5%) were confirmed by conventional RT-PCR. Twenty-three of the confirmed samples (59%) were sequenced. The most frequent strain detected was HCoV-OC43-like followed by NL63-like; only one sample was positive for HCoV-229E and one for HCoV-HKU1. Feline-like CoV strains were detected in three samples, representing possible evidence of interspecies transmission or a new human strain. Seventeen percent of the coronavirus positive samples were also positive for other respiratory viruses, such as Respiratory Syncytial Virus (RSV), Parainfluenza 2 and 3, and Rhinovirus. Thus, HCoV-OC43, NL63, HKU1 and new feline-like strains were circulating in Arkansas in 2010. HCoV was the sole respiratory virus detected in 16% of the patients who showed acute respiratory symptoms with negative diagnoses for influenza virus.

## Introduction

Acute respiratory viruses cause substantial morbidity and mortality worldwide. Most respiratory viral infections induce self-limiting disease. However, the disease range can vary from common cold, croup, and bronchiolitis to pneumonia, with an array of possible etiological agents, such as parainfluenza, influenza, RSV, adenovirus, rhinovirus, bocavirus, human metapneumovirus and coronavirus [1,2]. Coronaviruses (CoV) are responsible for a broad spectrum of diseases, including respiratory and enteric illnesses, in humans and animals [3]. Human coronaviruses (HCoV) were identified as the cause of acute respiratory tract disease in the early 1960’s [4], but their correlation with mild respiratory tract infection outweighed the importance of severe forms of the infection [5]. The emergence of SARS-CoV in humans in 2003 increased scientific interest in CoVs and emphasized the ability of highly pathogenic CoVs, most importantly those of animal origin, to infect humans. Consequently the importance of monitoring circulating coronavirus strains in humans has been reemphasized with the emergence of SARS and Middle East Respiratory Syndrome (MERS) CoV in humans [6,7].

The family Coronaviridae was recently subdivided into four genera according to their antigenic and genetic characteristics: Alphacoronavirus, Betacoronavirus, Gammacoronavirus and Deltacoronavirus (http://ictvonline.org/virusTaxonomy.asp?version=2012). Alphacoronavirus (HCoV-229E and HCoV-NL63) and Betacoronavirus (HCoV-OC43, SARS-CoV, HCoV-HKU1 and HCoV-MERS) infect a wide range of mammals [4,7–11], whereas members of the genus Gammacoronavirus and Deltacoronavirus usually infect birds [3], although a Gammacoronavirus was isolated from a Beluga whale [12]. Feline CoV, an Alphacoronavirus, infects wild and domestic cats causing mild enteritis. However, a lethal systemic disease known as feline infectious peritonitis (FIP) is also associated with FCoV. Feline CoV is closely related to CCoV, TGEV and human coronavirus HCV-229E, especially the Feline aminopeptidase N, which can be used as a functional receptor by these viruses [13].

The CoVs have a positive-sense, single-stranded RNA genome of 27–32 Kb. Nine to fourteen open reading frames (ORF) have been identified in the CoV genome. ORF1a and ORF1b encode the highly conserved replicase complex [14]. Most RT-PCR assays described in the literature to screen for CoV target the ORF1b region [15]. CoVs show a high frequency of nucleotide mutation and RNA recombination through copy-choice mechanism which, associated with broad receptor and co-receptor usage allow the virus to increase pathogenicity and possibly shift its host range [16].

Before the SARS-CoV outbreak, only two HCoV respiratory strains, HCoV-229E and HCoV-OC43 [4,8], had been described. Due to the increased interest highlighted by the SARS outbreak, three new strains were described afterwards; HCoV-NL63 [9], HCoV-HKU1 [10] and HCoV-MERS [17]. This study aimed to investigate the circulation of human respiratory CoV and to determine the genetic variability of HCoV in Arkansas.

## Materials and Methods

### Specimens

Two hundred nasal swabs provided by the Arkansas Department of Health were collected from March to July 2010 for influenza surveillance from patients showing respiratory disease symptoms. All patients exhibited influenza-like symptoms consisting of fever, chills, body aches, runny nose and/or nasal congestion. Patients had a median age of 56 years (ranging from <1 to 96 years of age); 47.5% were male and 52.5% female. No further information was available on the patients. Samples were collected in 3 ml of sterile viral transport medium, centrifuged, aliquoted and stored at −80°C until testing. All samples were screened using the CDC Human Influenza Virus Real Time RT-PCR Diagnostic Panel (cat# KT0096) and the CDC rRT-2009 A(H1N1) pdm Flu Panel (catalog #FLUSW04), by the Arkansas Department of Health. Only Influenza virus negative samples were selected for this study. All human samples were obtained by the Arkansas Department of Health according to institutional policies and Federal guidelines.

### Viral RNA extraction

Viral RNA was extracted from 90 µl of nasal fluid using the MagMax™ viral RNA isolation kit (Cat#1836) and the MagMAX™ Express Magnetic Particle Processor (Cat# 4400074, Applied Biosystems/Ambion, Austin, TX) according to manufacturer’s instructions. Extracted RNA samples were either analyzed immediately by real time RT-PCR or stored at −80°C until use.

### Real time RT-PCR (qRT-PCR)

Multiplex, real-time one-step RT-PCR was performed using the Rotor-Gene Multiplex RT-PCR kit (Qiagen, Valencia, CA) according to the manufacturer’s instructions. The reactions were carried out in duplicate, using primers and Locked Nucleic Acid (LNA) FAM, ROX and Cy5 labeled fluorescent overlapping probes (Integrated DNA Technology, Coralville, IA) for the open reading frame (ORF) 1b region of the CoV genome [15,18]. The ORF1b encodes the replicase complex of CoV (Table 1). A synthetic oligonucleotide complementary to the probe was used to generate a standard curve [18]. Primers and probes for 18S RNA (Cat# 4308329, Applied Biosystems, Foster City, CA) were used as internal controls [19]. Because of the availability and high identity to human CoV OC43 strain, the cell culture-adapted BCoV strain 88 RNA, IBV RNA and HCoV229E were used as positive controls. RNA from mock-infected cell supernatant was used as a negative control and RNAse-free water was used as a non-template control. The detection limit of the test was determined to be 2×10^2^ copies per ml.

For all assays, 5 µl of RNA was transferred to a Qiagen Rotor-Gene strip tube containing 15 µl of the Rotor Gene™ Multiplex qRT-PCR mix (Qiagen, Valencia, CA), with a total reaction volume of 20 µl, containing 10 µl of 2× Rotor-Gene Multiplex RT-PCR master mix, 800 nM of each primer, 200 nM of each probe, and 0.2 µl of Rotor-Gene RT mix. The thermal cycling conditions consisted of reverse transcription at 50°C for 30 min, 95°C for 10 min, 5 touchdown amplification steps of 94°C for 30 sec and 56°C for 30 sec, decreasing by 2°C every second cycle down to 48°C for 30 sec, and then 50 cycles of 94°C for 30 sec and 46°C for 60 sec. Amplification was detected using a Rotor Gene Q6-plex machine from Qiagen.

### One-step RT-PCR

RNAs positive for CoV by qRT-PCR were analyzed by one-step RT-PCR, using degenerate primers for the ORF1b [20] and specific primers (Table 1) for the spike and nucleocapsid genes of human alphacoronavirus (NL63 and 229E) and betacoronavirus (OC43 and HKU1). The specific primers were designed with the aid of the web-available software Primer 3 Plus or published elsewhere [20–23].

RT-PCR was performed using the One-step RT-PCR kit (Cat#210212, Qiagen, Valencia, CA) according to the manufacturer’s instructions. The reactions had a final volume of 25 µl, containing 5 µl of 5× Qiagen One Step RT-PCR Buffer, 600 nM of each primer, 400 µM of dNTP mix, 10 U of RNAseOUT (Invitrogen) and 1.0 µl of Qiagen One Step RT-PCR Enzyme Mix. The thermal cycling conditions were: reverse transcription at 50°C for 60 min, Hot-Start Taq DNA polymerase activation at 95°C for 15 min; 40 cycles of denaturation at 94°C for 30 sec, annealing at 48–50°C for 30 sec and extension at 72°C for 30 sec; followed by a final extension step at 72°C for 10 min.

The PCR products were separated on 1.5% agarose gel electrophoresis, stained with GelRed™ (Phenix). Bands of expected size were excised from the gel and purified using QIAquick Gel Extraction kit (Qiagen, Valencia, CA). Purified PCR products were sequenced directly or cloned into the pCR4.1 vector for confirmation and identification of the CoV strains.

### Virus amplification using cell culture

Samples positive for human CoV by qRT-PCR but negative by RT-PCR, using the ORF1b primers, were passed in HRT-18 (human rectal tumor - ATCC cat# CCL-244), Vero (African green monkey kidney - ATCC cat# CCL-81) and/or MRC-5 (human fetal lung – ATCC cat# CCL-171) cells in an effort to isolate and further amplify CoV, if present. Briefly, cells were grown in supplemented Advanced-Minimum Essential Medium (Ad-MEM, Invitrogen), containing 1% antibiotic-antimycotic (Gibco) and 5% fetal bovine serum (FBS), or in Dulbecco’s Modified Eagle Medium (DMEM, Invitrogen), supplemented with 1% antibiotic-antimycotic and 10% FBS, for 2–5 days. Before infection, cells were rinsed and incubated with Minimum Essential Medium (MEM, Invitrogen) without FBS for 1–3 h at 37°C and 5% CO2. T25 cm2 flasks (Nunc, Inc.) were inoculated with 200–300 µL of filtered (0.22 µm) nasal swab fluids diluted 1:25 in MEM. After 1 hour of incubation at 37°C, the supernatant was removed and replaced with FBS free MEM containing 0.15 µg/ml of trypsin (Sigma cat# T1426). Cells were frozen when 50 to 80% cytopathic effects were evident or after 14 days of incubation. After a cycle of freezing and thawing, cell lysates were centrifuged for 15 min at 2,500 × g at 4°C, and the supernatants were stored for subsequent inoculation or used for viral RNA extraction. Samples were passaged until a RT-PCR band was detected, or for up to 10 blind passages in each cell line.

### Sequencing of RT-PCR products and phylogenetic analysis

Sequencing of RT-PCR products was performed using the Applied Biosystems (ABI-3100) automated sequencing system with the ABI Big Dye™ Terminator sequencing kit, version 3. Sequences obtained were submitted to a similarity search using BLAST and phylogenetic analysis.

Nucleotide sequences based on a 251 bp fragment of the polymerase region of CoV, 390–412 bp products of the spike region and 247–462 bp of the nucleocapsid region were compared to sequence of reference strains using Clustal W [24]. Phylogenetic dendrograms were constructed for each region using the neighbor-joining method supported with a bootstrap test of 1000 replicates in MEGA 5 software [25].

### Detection of other respiratory viruses

Coronavirus positive samples were also screened for the presence of Parainfluenza 1, 2 and 3, Respiratory Syncytial virus, rhinovirus and adenovirus, using multiplex PCR kits from Maximbio (Cat# MP-70177 and MP-70202). Briefly, cDNA was generated using SuperScript III RT (Invitrogen) and random hexamers. The multiplex PCR reaction had a final volume of 25 µl, containing 12.5 µl of 2× MPCR buffer, 2.5 µl of 10× multiplex primers, 2.5 U of Taq DNA polymerase and 2.5 µl of cDNA. The thermal cycle conditions were 2 cycles of 96°C 1 min, 55°C for 4 min; 35 cycles of 94°C for 1 min, 55°C for 2 min, followed by a final extension step at 70°C for 10 min. The PCR products were separated on 2% agarose gel electrophoresis stained with GelRed (Phenix). Bands of expected size were compared to the positive controls supplied by the manufacturer.

### Statistical analysis

The incidence of coronavirus according to age distribution and gender was analyzed using Chi-square. The analyses were conducted using SigmaPlot 11.0 (Systat Software, Inc).

## Results

### Real time qRT-PCR and RT-PCR detection

When qRT-PCR was used to screen the samples for CoV, 79 out of 200 (39.5%) samples were positive. Thirty-five out of 200 (17.5%) were confirmed to be positive for coronavirus by RT-PCR, using primers for the ORF1b, N and/or S regions (Tables 2 and 3). Twenty-four samples were amplified using primers specific for the N or S regions of OC43, 5 for NL63, 3 for HKU1, 1 for 229E and 1 for feline-like (Table 3). Among those samples, 6 were amplified only after the filtered nasal aspirates were passed into cell culture; 5 of them were passed into Vero cells and 1 was passed into HRT-18 cells. All of them were amplified using primers to the S region of OC43.

The age distribution of coronavirus positive samples confirmed by RT-PCR is shown in Table 2. High incidence of respiratory CoV occurred in children up to 10 years of age (17.1%) and in adults above 40 years of age (11.4–25.7%). Gender distribution of coronavirus positive samples confirmed by RT-PCR showed a higher incidence of positive samples in females (13.0%) versus males (4.5%) (P<0.05).

### Sequence analysis of CoV strains

**Figure 1:**
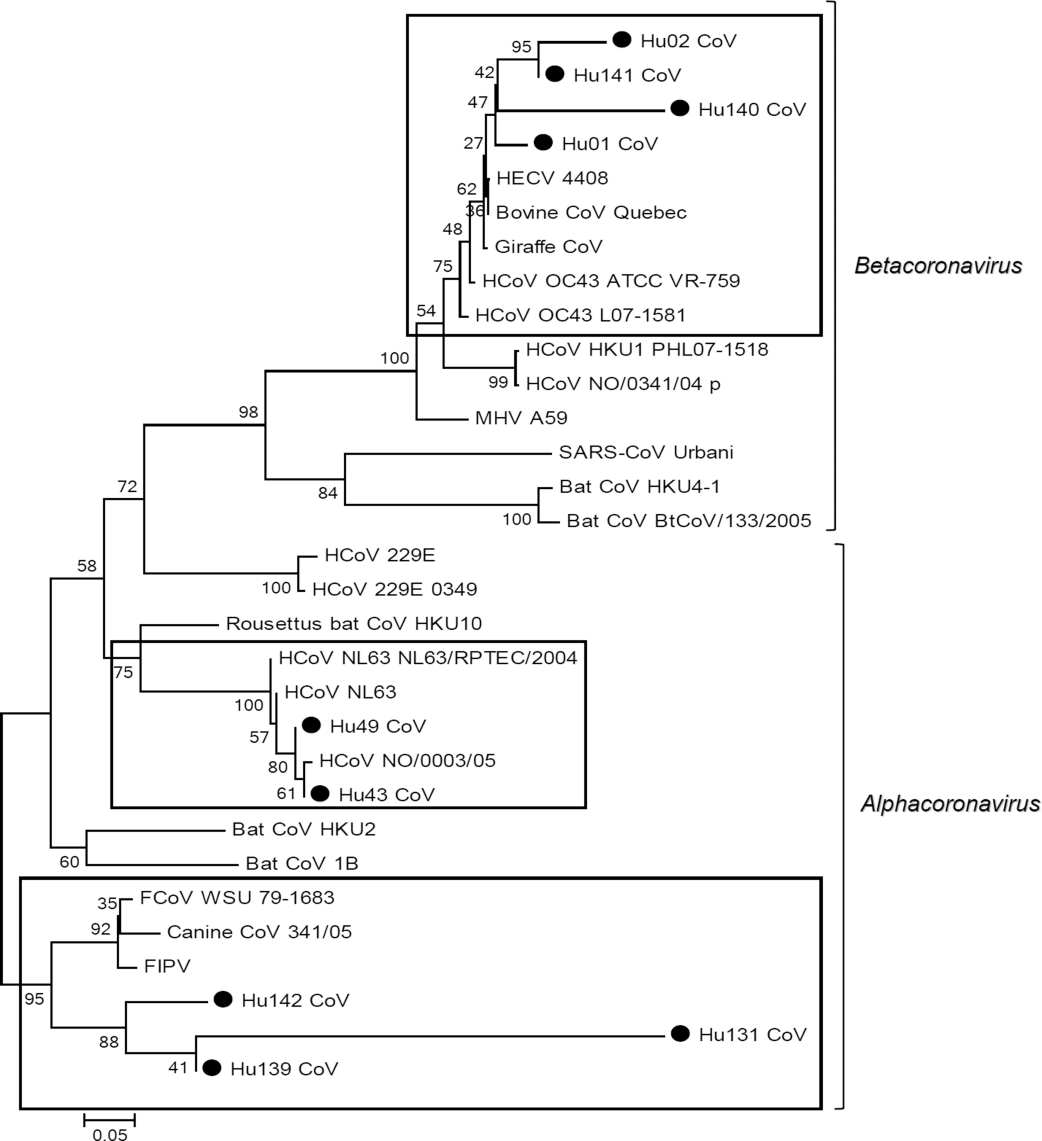
Phylogenetic dendrogram of the partial (251 bp) sequence of ORF1b region. The evolutionary history was inferred using the Neighbor-Joining method [32]. The percentages of replicate trees in which the associated taxa clustered together in the bootstrap test (1000 replicates) are shown next to the branches [33]. The tree is drawn to scale, with branch lengths in the same units as those of the evolutionary distances used to infer the phylogenetic tree. The evolutionary distances were computed using the Maximum Composite Likelihood method [34] and are in the units of the number of base substitutions per site. Evolutionary analyses were conducted in MEGA5 [25]. ● denotes samples from this study.

**Figure 2:**
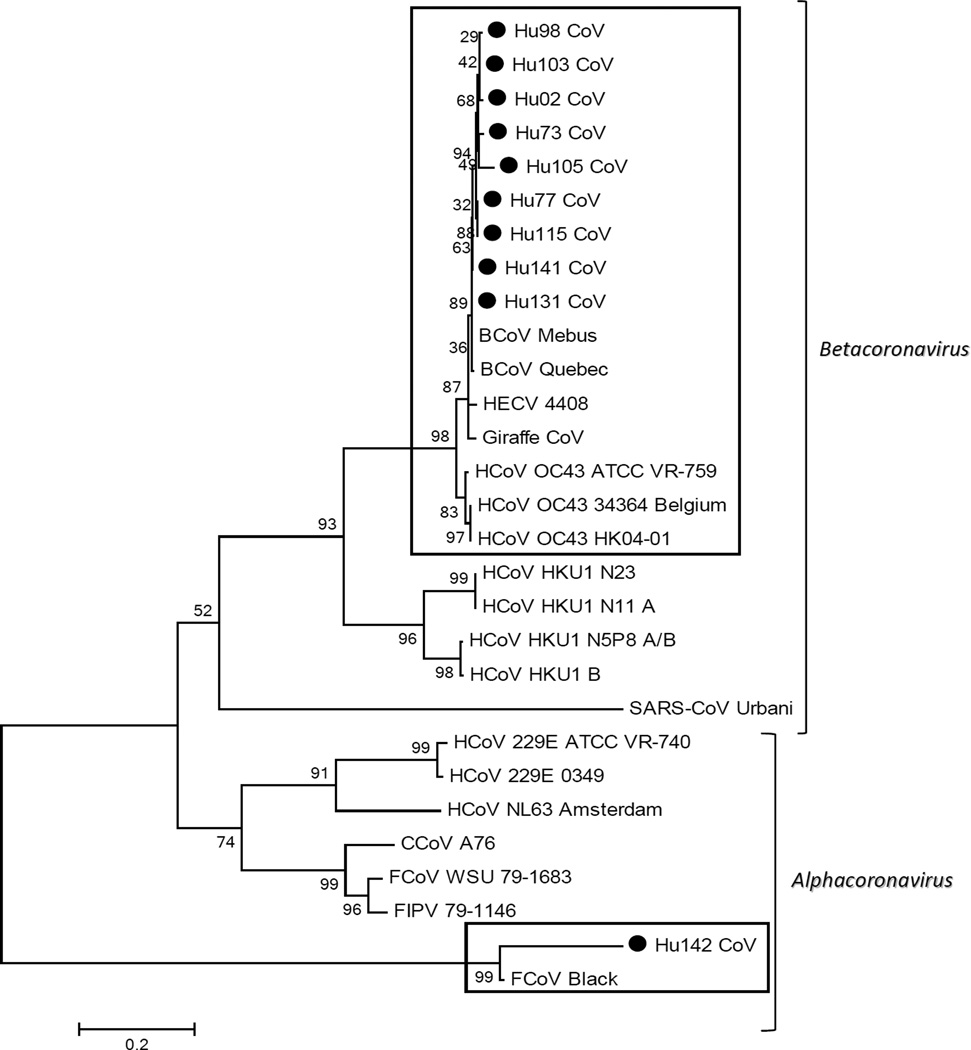
Phylogenetic dendrogram of the partial (460 bp) sequence of the Spike region. The evolutionary history was inferred using the Neighbor-Joining method [32]. The percentages of replicate trees in which the associated taxa clustered together in the bootstrap test (1000 replicates) are shown next to the branches [33]. The tree is drawn to scale, with branch lengths in the same units as those of the evolutionary distances used to infer the phylogenetic tree. The evolutionary distances were computed using the Maximum Composite Likelihood method [34] and are in the units of the number of base substitutions per site. Evolutionary analyses were conducted in MEGA5 [25]. ● denotes samples from this study.

**Figure 3:**
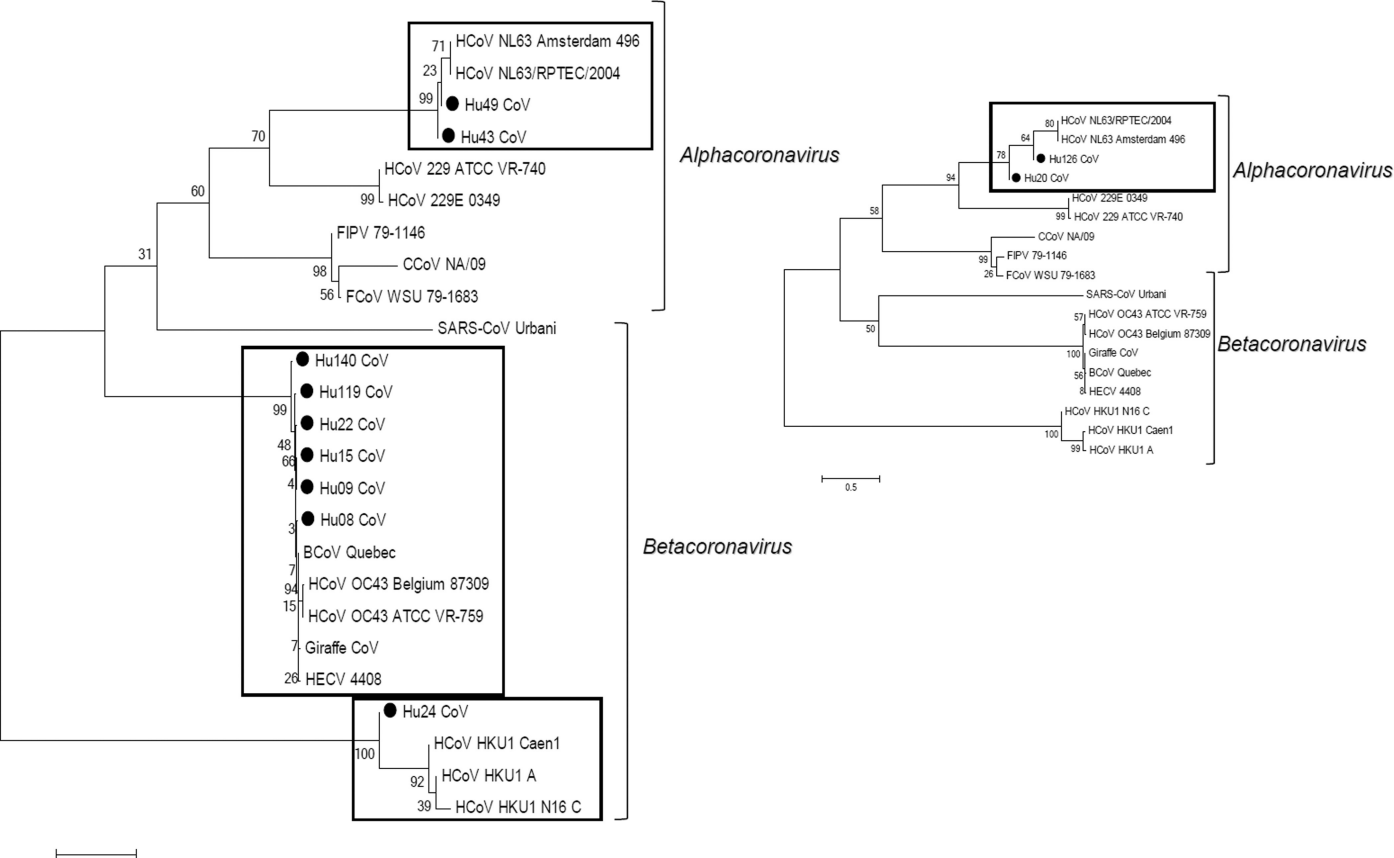
Phylogenetic dendrogram of the partial (460 bp) sequence of the nucleocapsid region. Because only 100–140 bp fragments were obtained for Hu20&126 samples, they were analyzed separately and are shown on a smaller subtree. The evolutionary history was inferred using the Neighbor-Joining method [32]. The percentages of replicate trees in which the associated taxa clustered together in the bootstrap test (1000 replicates) are shown next to the branches [33]. The tree is drawn to scale, with branch lengths in the same units as those of the evolutionary distances used to infer the phylogenetic tree. The evolutionary distances were computed using the Maximum Composite Likelihood method [34] and are in the units of the number of base substitutions per site. Evolutionary analyses were conducted in MEGA5 [25]. ● denotes samples from this study.

**Figure 4:**
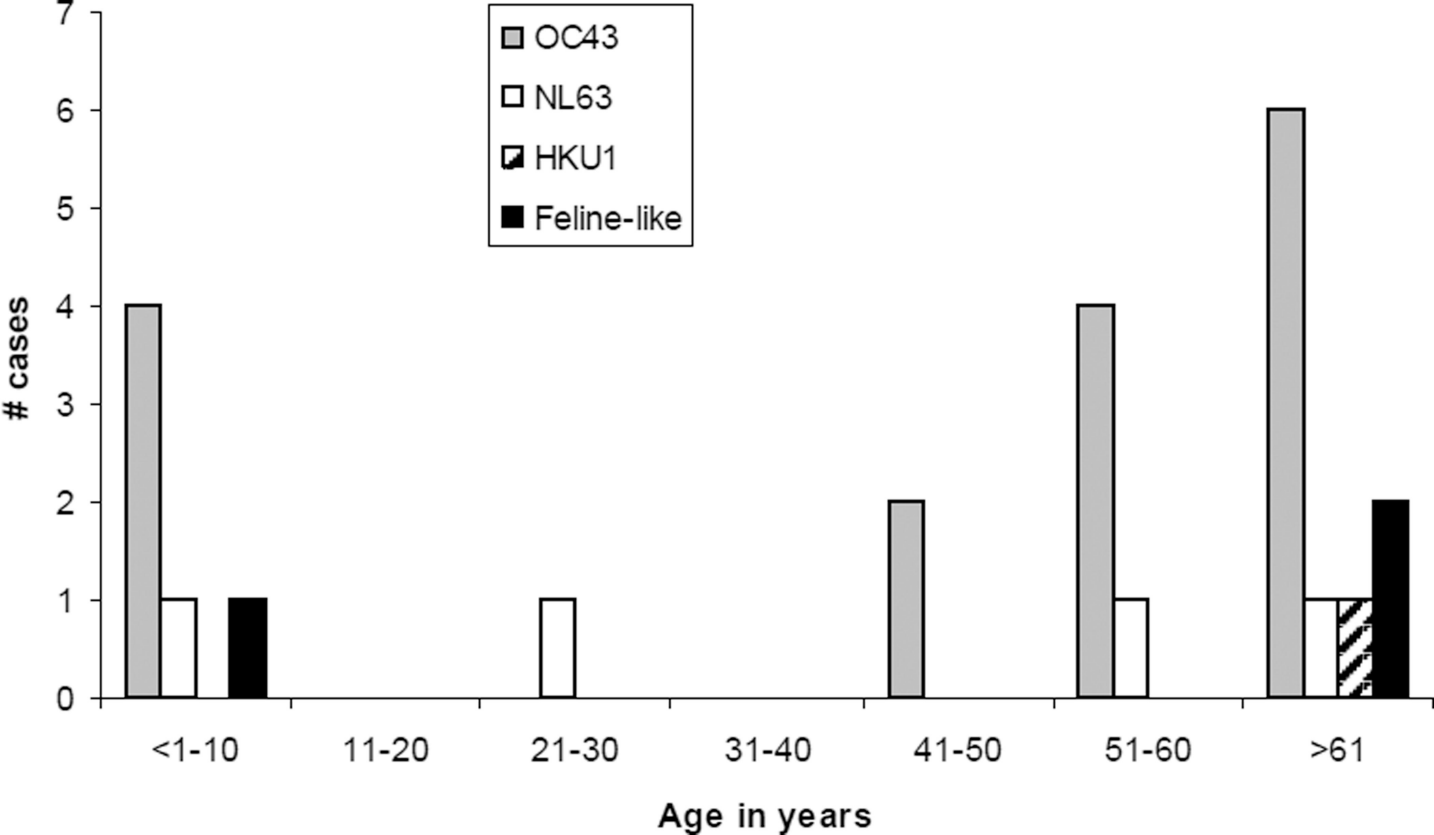
Strain distribution of respiratory coronavirus in Arkansas by age group and number of cases.

Because of poor template quality, not all samples could be amplified with the same primers. Twenty-three CoV positive out of 35 (65.7%) samples detected by RT-PCR were successfully sequenced and analyzed using BLAST software and phylogenetic analysis (Table 4, Figures 1–4). A phylogenetic tree was constructed by comparing the sequences from 251 bp fragments of ORF1b of 9 samples with reference strains available from the GenBank database, showing that 2 CoV positive samples were closely related to NL63, 4 were closely related to OC43 (and HECV 4408 and BCoV) and 3 samples were closely related to Feline CoV (ID to FIPV=81.9%, 79.8 and 65.8%, respectively).

The sequence of RT-PCR products within the spike protein region revealed 9 samples that shared highest identity to HCoV-OC43, HECV 4408 and BCoV. Among the 3 feline–like samples (based on ORF1b phylogenetic analysis), two were amplified and sequenced using primers to the S region, one was shown to share the highest identity with the OC43 strain (95.9% identity) and another shared the highest identity to feline CoV (83.8% identity) (Figure 2).

The sequence of RT-PCR products within the nucleocapsid region of 11 samples revealed that 6 samples shared high identity with OC43, 4 clustered together with NL63 and 1 clustered with HKU1 (Figure 3). One sample was amplified with primers to the nucleocapsid region specific to strain 229E, but no sequence was obtained from it. The age distribution of the strains identified showed that OC43-like strains were detected in individuals younger than 10 years or older than 40. The NL63-like strains and, interestingly, the feline-like CoVs were detected only in the youngest and the oldest patients (Figure 4).

The sequences from this study were deposited in GenBank under accession numbers KF524838 to KF524862.

### Other respiratory viruses

All respiratory samples positive for coronavirus by real-time RT-PCR were screened for other respiratory viruses. Among the real-time RT-PCR CoV positive samples, 13% were also positive for Respiratory Syncytial Virus, Parainfluenza 2 and 3, and Rhinovirus (Data not shown). Among the patients harboring respiratory CoVs, detected by qRT-PCR and confirmed by RT-PCR, six (17%) were co-infected with a second respiratory virus.

## Discussion

To characterize the HCoV circulating in Arkansas, we targeted patients showing respiratory tract infection symptoms, but negative for Influenza virus. We found that thirty five (detected by qRT-PCR and confirmed by RT-PCR) patients harbored respiratory CoVs and six (17%) of those patients were co-infected with a second respiratory virus. A three year study conducted in the UK found that 11 to 41% of the samples positive for CoV were also positive for other respiratory viruses [26], and CoV monoinfection was comparable to other viruses that have well established respiratory diseases. We also observed a trend in the gender distribution of the confirmed RT-PCR samples with a higher incidence in females compared to males. At this point, the significance of this finding is unclear, since a gender trend was not observed on the screened samples using the qRT-PCR and similar findings have not been reported.

A study by Meir et al. [27] using a real-time RT-PCR assay to detect infectious bronchitis virus (IBV) was shown to be 1 to 4 times more sensitive than RT-PCR targeting the S or N regions. We also observed a discrepancy between qRT-PCR and RT-PCR detection. In our study, a multiprobe qRT-PCR was 2-fold more sensitive than the combined RT-PCR assay against the ORF1b, N and S regions. We believe the RNA integrity was the key for this discrepancy, since we did not observe a correlation between RNA copy number and RT-PCR positivity; rather linked to product size (150bp vs 250–480bp).

The most prevalent strain detected was closely related to HCoV-OC43, occurring in patients younger than 10 or older than 40 years of age. The second most prevalent strain detected was closely related to NL63, which has been related to acute respiratory symptoms, mainly in children and immunocompromised individuals [28,29]. Previous studies (2007 and 2009) in the US on the prevalence of HCoV in respiratory tract infections of children reported different circulating strains by region. In the Rocky Mountain region, the predominant strains were NL63, OC43 and 229E, while in the Pacific Northwest, HKU1 was predominant [21,30]. In this study, we surveyed both children and adults and demonstrated that OC43 and NL63 CoVs were the predominant strains. In another study, using samples from Rochester, NY, Nashville, TN and Cincinnati, OH, the findings were similar to our study, in which the OC43 was the most prevalent strain, followed by NL63 and HKU1, and 229E was the least prevalent [31]. Prill et al. [31] reported that OC43 tended to circulate from fall to winter and NL63 from winter to spring, while 229E was not detected in the first four years of the study and only one cluster of HKU1 during a single winter. In a study by Kuypers et al. [30], HKU1 was more likely to occur from October to February and 229E from December to June. In a study by Domiguez et al. [21], HKU1 was more likely to occur from February to May and 229E from February to June. Based on the distribution of HKU1 and 229E the studies of Kuypers et al. [30] and Dominguez et al. [21], we should have been able to detect both strains if they had circulated with similar seasonality in Arkansas; however, only one case of 229E and one case of HKU1 were detected.

We also detected three isolates that showed high identity to feline enteric coronavirus (Alphacoronavirus), one from a child and two from adults over 60 years of age. However, a 400 bp fragment from the S region of one of these samples revealed an OC43-like spike region, suggestive of feline and OC-43 coronavirus co-infection, since recombination between alpha and betacoronaviruses has yet to be documented. The virus load of the diluted nasal swabs for the three feline-like strains ranged from 3×10^4^ to 1×10^5^ RNA copies per ml (data not shown), which was insufficient for next generation sequencing. We are currently trying to cell adapt the feline-like strains to amplify the virus load for full genome sequencing.

Despite the self-limiting aspect of interspecies transmission, the possibility of adaptation and generation of recombinant viruses may pose a threat for epidemics or pandemics. Moreover, continuous surveillance is further justified by the current emergence of a novel coronavirus strain in the Middle East (MERS CoV) [17] and by avian influenza infections occurring in the human population.

In conclusion, OC43, NL63 strains and a new feline-like strain circulating in Arkansas may have been associated with the acute respiratory symptoms observed in patients negative for influenza virus. Whether the feline-like coronavirus strain represents a direct case of interspecies transmission or a new human strain is not clear at this point. The severity of symptoms associated with the CoV infection and/or other respiratory viruses may have encouraged patients to seek clinical diagnosis of influenza infection.

## Figures and Tables

**Table 1:** Primer and probe sequences used for RT-PCR or qRT-PCR.

Gene position range	Sequence (5’→3’)	Product (bp)	Source
RT-PCR			
ORF1b* 14900–15150	F-ACWCARHTVAAYYTNAARTAYGC R-TCRCAYTTDGGRTARTCCCA	250	[19]
NL63-Spike 21169–21558	F-CGTTTAACTTGTTTATGGCCTG R-ATAAAACTGACCAGTTCTAGCAA	390	This study
OC43-Spike 26091–26490	F-GTGCTGCATTTGTCTGTGGT R-AGCAGTGGAGGCAACACTTT	400	This study
229E-Spike 20420–20835	F-TACCCTCCGACTTTGCATTC R-TAGTGCACCCACAAAAGCAC	415	This study
HKU1-Spike 25497–25923	F-TATGCAGCTTGCCATGACTT R-CAGTAGCGGCTGTGGTGTAA	426	This study
Feline-Spike 24214–24589	F-GTTTCAACCTAGAAAGCCTCAGAT R-CCACAGATACCAAGGCC	375	[22]
NL63-Nucleocp 26211–26534	F-AGATGAGCAGATTGGTTATTGG R-GAAGAGTCTCGTGAGTTGTTAC	324	[21]
OC43-Nucleocp 29325–29769	F-TGCCTATTGCACCAGGAGTC R-TCAGCCATGTCAGGTGTTAC	445	[20]
229E-Nucleocp 25862–26342	F-AGTCGCGGTCGTGGTGAATC R-CAGTGTTGCCTGACTCTTTG	480	[20]
HKU1-Nucleocp 29342–29588	F-GCTCCTACACCAGGTGCTTT R-CACCTCTTTTACGCTGAGGTTT	247	This study
qRT-PCR**			
ORF1b	F-TGATGATGSNGTTGTNTGYTAYAA R-GCATWGTRTGYTGNGARCARAATTC	154	[14,17]
Probes			
Probe I	[Fam]TTGTATTATCAGAATGGYGTSTTYATG[BHQ]		[14]
Probe II	[Rox]TGTGTTCATGTCWGARGCWAAATGTTGC[ECLIPSE]		[14]
Probe III	[Cy5]TCTAARTGTTGGGTDGA		[14]

* Positions may vary depending on the strain

** qRT-PCR primers and probes vary positions from 14405 to 16013, depending on the strain. LNA positions are underlined.

**Table 2:** Samples and age distribution of coronavirus in influenza negative human respiratory samples during the 2009–2010 influenza season in Arkansas.

Age	Total (%)	qRT-PCR (%)	RT-PCR (%)	Sequenced
≤10	19 (9.5)	9/79 (11.4)	6/35 (17.1)	5
11–20	3 (1.5)	1/79 (1.3)	0/35 (0.0)	-
21–30	17 (8.5)	6/79 (7.6)	2/35 (5.7)	1
31–40	16 (8.0)	6/79 (7.6)	0/35 (0.0)	-
41–50	27 (13.5)	10/79 (12.6)	4/35 (11.4)	2
51–60	30 (15.0)	14/79 (17.7)	8/35 (22.9)	5
61–70	36 (18.0)	16/79 (20.2)	6/35 (17.1)	5
≥71	52 (26.0)	17/79 (21.5)	9/35 (25.7)	5
**Gender**				
Female	105 (52.5)	41 (20.5)	26 (13.0)*	16
Male	95 (47.5)	38 (19.0)	9 (4.5)	7
**Total**	**200**	**79/200 (39.5)**	**35/200 (17.5)**	**23**

* Males vs Females = P<0.05

**Table 3:** RT-PCR results using specific primers for CoV spike and/or nucleocapsid genes.

Alphacoronavirus			Betacoronavirus		
NL63	229E	Feline-like	OC43	HKU1
5/35* (14.3%)	1/35 (2.9%)	1/35 (2.9%)	24/35 (68.6%)	3/35 (8.6%)

* All 79 qRT-PCR positive specimens were further screened using specific primers, 34 were positive using spike and/or nucleocapsid primers, and one was only amplified using ORF1b primers

**Table 4:** CoV strains identified by sequence analysis of the product amplified using the RT-PCR ORF1b, N or S primers.

Alphacoronavirus		Betacoronavirus	
NL63	Feline CoV	OC43	HKU1
4/23 (17.4%)	3*/23 (8.7%)	16/23 (69.6%)	1/23 (4.3%)

* Samples sequenced by multiple products are reported once. One sample, sequenced as OC43 for the spike region and feline CoV for ORF1b, was reported as Feline CoV.
